# Plagiarism in the article Emphysematous cystitis and emphysematous pyelitis: a clinically misleading association. Mustapha Ahsaini et al. The Pan African Medical Journal. 2013;16:18. (doi:10.11604/pamj.2013.16.18.2505)

**DOI:** 10.11604/pamj.2015.21.288.7763

**Published:** 2015-08-18

**Authors:** PanAfrican Medical Journal

**Affiliations:** 1 The Pan African Medical Journal, Yaoundé, Cameroun

**Keywords:** Plagiarism, Retraction, PAMJ

## Abstract

This retracts article *Emphysematous cystitis and emphysematous pyelitis: a clinically misleading association. Mustapha Ahsaini, Amadou Kassogue, Mohammed Fadl Tazi, Anas Zaougui, Jalal Edine Elammari, Abdelhak Khallouk, Mohammed Jamal El Fassi, My Hassan Farih. The Pan African Medical Journal. 2013;16:18. (doi:10.11604/pamj.2013.16.18.2505)*.

## Retraction

We hereby inform to our readership of the retraction of the article *Emphysematous cystitis and emphysematous pyelitis: a clinically misleading association(doi:10.11604/pamj.2013.16.18.2505) by Mustapha Ahsaini, Amadou Kassogue, Mohammed Fadl Tazi, Anas Zaougui, Jalal Edine Elammari, Abdelhak Khallouk, Mohammed Jamal El Fassi and My Hassan Farih* of the Department of Urology, University Hospital, Center Hassan II, Fes, Morocco [1]. From our investigation, it clearly appears that the majority of the contents of the article by Mustapha Ahsaini and al. published in PAMJ in September 17, 2013 was plagiarized from the article Emphysematous cystitis of the diabetic patient. 2009 Aug; 1(3): 114-116. PMCID: PMC3364639 by Affes Nejmeddine, Bahloul Atef, Dammak Youssef, Beyrouti Ramez, and Beyrouti Mohamed Issam, published in North American Journal of Medical sciences [2]. Therefore, we withdraw this article of the medical literature in accordance with the guidelines and best editorial practices of the Committee on Publication Ethics. We apologize to our audience for this unfortunate situation.

## References

[1] Mustapha Ahsaini, Amadou Kassogue, Mohammed Fadl Tazi, Anas Zaougui, Jalal Edine Elammari, Abdelhak Khallouk, Mohammed Jamal El Fassi, My Hassan Farih (2013). Emphysematous cystitis and emphysematous pyelitis: a clinically misleading association. The Pan African Medical Journal..

[2] Affes Nejmeddine, Bahloul Atef, Dammak Youssef, Beyrouti Ramez, Beyrouti Mohamed Issam (2009). Emphysematous cystitis of the diabetic patient. N Am J Med Sci..

